# Mr. Davies, in Reply to Dr. Squire

**Published:** 1800-06

**Authors:** H. Davies

**Affiliations:** Piccadilly


### *To the Editors of the Medical and Phyſical Journal.*

Gentlemen,

I Obſerved in the laſt Number of your Journal, ſome "Remarks on the expulſion and extraction of the placenta after delivery," by Dr. Squire; to which, he ſays, "he was induced from the peruſal of two communications on the ſubject;" wherein, "he thought it a duty he owed to ſociety to prevent, as much as might be in his power, the mode of treatment therein deſcribed."

Since he has involved *two* correſpondents, equally "recommending a mode of practice ſo oppoſite to rules laid down by by the ableſt and moſt experienced men of the preſent time," and, withal, "ſo dangerous in its tendency," I can only be reſponſible for *one*, and think it equally my duty to refute a charge ſo palpably erroneous, and ſo injurious to medical reputation; for, although the doctrine he reprobates is ſtill maintained by ſome reſpectable practitioners, and, in ſome caſes, perhaps is indiſpenſable; yet I am convinced, from repeated obſervations, that the contrary mode of treatment is, for the moſt part, the moſt eligible.

How the Doctor came to confound my ſentiments with thoſe of Mr. Peck's, on the "Expediency of the *early* Delivery of the Placenta," at all events, I cannot poſſibly account for. For, the principal deſign of publiſhing my caſe was to recommend the *contrary* practice, unleſs imminent danger ſupervened; which induced Mr. P. to comment upon it in a former Number of your Journal; and, if in the management of my caſe alluded to, it appeared that I deviated from the ſentiment, the neceſſity aroſe from the appearance of ſymptoms which portended danger, if I had longer delayed to proceed in the manner deſcribed.

As to the Doctor's obſervations on my treatment of it, I am not at all concerned to notice; they might ſerve to amuſe him in writing, and they do not offend me in reading them: And, as to the *liberality* of his ſentiment in aſſerting that the ſubjects of his remarks, were the "erroneous opinions of men who have had no experience," likely to "miſlead the ignorant and unwary," I ſhall ſuffer it to prove its own confutation, only begging leave to obſerve, reſpecting medical "experience," that as ample a ſhare of it falls, ſometimes, to the lot of private practitioners, as certain public teachers.

I now take leave of the Doctor and his Remarks. Wiſhing him all poſſible ſucceſs in his intended publication on the ſame ſubject, to which, perhaps, the Remarks are a kind of prelude; and, that every future attempt to ſerve the general cauſe may be attended with every beneficial effect. I am,

GENTLEMEN,

Your's, reſpectfully,

H. DAVIES.

